# Confronting TB/HIV in the era of increasing anti-TB drug resistance

**DOI:** 10.1186/1758-2652-11-6

**Published:** 2008-11-06

**Authors:** Jeremiah Chakaya, Haileyesus Getahun, Reuben Granich, Diane Havlir

**Affiliations:** 1 Centre for Respiratory Diseases Research, Kenya Medical Research Institute, Africa; 2 Stop TB Department, World Health Organization, Geneva, Switzerland; 3 HIV Department, World Health Organization, Geneva, Switzerland; 4 University of California, San Francisco, USA

## Abstract

HIV associated TB is a major public health problem. In 2006, it was estimated that there were over 700,000 people who suffered from HIV associated TB, of whom about 200, 000 have died. The burden of HIV associated TB is greatest in Sub-Saharan Africa where the TB epidemic is primarily driven by HIV. There has been steady progress made in reducing the burden of HIV in TB patients with an increasing number of TB patients tested for HIV and provided with cotrimoxazole preventive therapy (CPT) and anti-retroviral treatment (ART). Less progress is being made to reduce the burden of TB in people living with HIV. The number of HIV infected persons reported to have been screened for TB was less than 1% while Isoniazid preventive therapy was reported to have been provided to less than 0.1% of eligible persons in 2006. A major push is urgently needed to accelerate the implementation of three important interventions. The three are Intensified TB Screening (ICF) among people living with HIV, the provision of Isoniazid Preventive Therapy (IPT) and TB Infection Control(IC). These interventions are best carried out by HIV control programmes which should therefore be encouraged to take greater responsibility in implementing these interventions.

## Burden of HIV associated TB

It is estimated that two thirds of the world population, or nearly two billion people, is infected with the tuberculosis bacillus[1] while about 33 million people were living with HIV by the end of 2007[2]. The convergence of the two epidemics affects about 11 million people. Tuberculosis is the most common HIV related infection and the most common cause of death in HIV infected persons. The HIV epidemic is the major factor responsible for the dramatic increase in TB incidence that has been witnessed in Sub-Saharan Africa over the last decade [3]. Even though Sub -Saharan Africa, with about 85% of the estimated 709,000 HIV associated TB infections in 2006, bears the brunt of TB/HIV, other "hot spots" for HIV associated TB include India and Brazil, which had 3.3% and 1% of the global burden respectively in 2006[1]. The TB and HIV epidemics are so intricately intertwined in Sub-Saharan Africa that the prevention and care of one must be linked with the prevention and care of the other [4].

HIV influences TB by increasing the risk of reactivation of latent TB infection, rapid progression of new TB infection to disease and recurrent disease from both re-infection and relapse [5]. Other than for differences in magnitude, TB is the most common opportunistic infection that is observed in the first three months of initiation of combined ART in both Sub-Saharan Africa and the industrialized world of North America and Europe [6,7]. Tuberculosis threatens the gains being made in the provision of ART to people living with HIV.

## Interventions for HIV associated TB

In 2004 the World Health Organization published the interim policy for TB/HIV collaborative activities, a comprehensive guide for the implementation of TB/HIV collaborative activities, which highlights twelve key activities whose full implementation is expected to lead to a significant impact on the burden of TB/HIV [8]. Since the publication of this document significant progress has been made regarding the establishment of mechanisms for collaboration and the reduction of the burden of HIV in TB patients. For example HIV testing of TB patients increased from 21,806, less than 1% of all notified cases, in 9 countries in 2002 to 687,174 patients, 12% of all notified cases, from 112 countries in 2006. In Africa 287, 945 patients (22% of all notified cases) were tested for HIV in 2006. Of the 186, 217 HIV infected TB patients detected in 2006, 146, 586 (78%) were placed on CPT and 66,601(41%) on ART [1]. In most countries CPT is largely provided at TB clinics while ART provision often implies referral from TB clinics to ART sites, leading to loss of patients and partly accounting for the low uptake of ART in HIV infected TB patients.

Although there is much work to be done to provide HIV counseling and testing to all TB patients, a number of countries have had exemplary performance in getting TB patients tested for HIV. These countries include Rwanda, Brazil, Malawi, Kenya and Botswana who achieved testing rates of more than 60% of notified TB cases by the end of 2006[1].

Some of the factors that have contributed to this performance include the establishment of appropriate national policies and guidelines, revision and/or adoption of new recording and reporting tools that are able to capture TB/HIV indicators, target setting at all levels of the health care system, appropriate financing and training, support of health care workers and providing services under one roof (table 1) [9,10]. These factors are greatly enhanced by stronger communication and collaboration between the TB and HIV control programmes.

**Table 1:** What is needed for nationwide scale up of TB/HIV Collaborative Activities

National policies and guidelines
Recording and Reporting tools

Collaboration and communication between TB and HIV control programmes

Leadership and commitment

Target setting at all levels of the health care system

Human resources: recruitment, training, support, task shifting

Financing

National Partnerships for results

Providing TB and HIV services under one roof

There has been less robust progress with activities intended to reduce the burden of TB in persons living with HIV. In 2006 only 0.9% of the estimated 33 million HIV positive people were screened for TB. Of the 314,934 HIV infected persons reported to have undergone screening for TB in 2006, 84,713 (27%) were found to have TB, implying a high yield of TB screening programmes for HIV infected persons. Only 0.1% (27,056 of 33 million HIV infected persons) of HIV infected persons were reported to have received Isoniazid Preventive Therapy in 2006. Of the 27,056 provided with IPT, 70% were in Botswana, the only country with a nationwide IPT programme [1]. While non-reporting or underreporting may be responsible for this rather grim scenario, there is real concern that not enough is being done to reduce the burden of TB in persons living with HIV.

To stimulate and accelerate action in this area WHO, and more specifically the HIV department of WHO, coined the term the 'Three I's' for Isoniazid Preventive Therapy (IPT), Intensified TB Case Finding (ICF) and TB infection Control (IC). In April 2008, WHO organized the 'Three I's' meeting where it was recognized that these three key interventions should be central to the scale-up of care and ART, given the threat posed by TB.

## Isoniazid preventive therapy

Several randomized clinical trials have demonstrated that TB can be effectively prevented using Isoniazid for 6–12 months in HIV negative persons [11]. As the HIV epidemic unfolded and TB was recognized to be a major problem in HIV infected persons, a series of studies designed to test the efficacy of Isoniazid in preventing HIV associated TB were carried out. The effects were similar to what had been observed in HIV non-infected persons. The incidence of HIV associated TB could be effectively reduced but IPT had no impact on all cause mortality. The beneficial effect of IPT was associated with only a slight increase in adverse drug effects. The protective effect diminishes with time and lasts one to three years on average [12]. While IPT has been found to have no effect on mortality of HIV infected adults, a South African study documented a significant effect on mortality of HIV infected children and the placebo arm of this study had to be prematurely terminated [13].

Having established the efficacy of IPT in preventing HIV associated TB, the TB community moved on to document feasibility of this intervention under "routine" programme conditions. The results were somewhat disappointing with high attrition rates and low treatment completion rates [14,15].

Despite the documented efficacy and feasibility of IPT for the prevention of HIV associated TB, this intervention has not yet been taken up on a large scale. This was probably the result of policy uncertainties as the earlier message by technical agencies including WHO only promoted TB preventive therapy for "personal protection" as opposed to a public health intervention designed to impact on the community incidence of TB. Additionally there were fears that TB preventive therapy programmes could expand Isoniazid resistance because of difficulties in confidently excluding active TB. There were also concerns about safety. The non involvement of HIV control programmes and communities, including the community of HIV infected persons, may also have contributed to the low uptake of this intervention.

Although IPT targets HIV infected persons who are not suffering from TB and are therefore not in TB control programmes, there were uncertainties over which programme, between the TB and HIV control programmes, should be responsible for this intervention. It is therefore not surprising that to date only Botswana has a countrywide TB preventive therapy programme which is nested within the TB control programme. The landscape has, however, completely changed since the time that the IPT feasibility studies were undertaken. There is a new push for IPT and greater engagement of HIV control programmes coupled with HIV activism in favor of IPT which is expected to lead to some progress in the provision of IPT. There is a very urgent need however for the development of appropriate recording and reporting tools to allow for the routine collection, collation and analysis of data related to the provision of IPT. We are hoping that these will form part of the pre-ART care register and be central to recording and reporting on HIV care delivery within HIV services.

## Anti-retroviral therapy and IPT

With the advent of combined ART it was observed that TB incidence fell in persons on ART in North America and Europe. The fall in TB incidence has been observed to be greater in persons with a higher baseline CD4 T cell count, a lower base line viral load and robust immunological and virological responses [16]. Similar observations have been made in South Africa [17]. Questions have therefore been raised about the added value of IPT in preventing TB in persons receiving ART. A recent retrospective study carried out in Rio D Janeiro, Brazil, suggests that the combination of ART and IPT has the greatest impact on TB incidence when compared to IPT or ART alone[18] and provides further impetus to provide IPT in all HIV infected persons irrespective of whether they are or are not on ART. This approach would conform with the observation that the risk of TB in HIV infected persons remains higher than that in non-HIV infected persons even when on ART[19].

## IPT and the risk of drug resistant TB

The recent outbreaks of MDR and XDR TB have reinforced the fear of expansion of isoniazid resistance. Expansion of Isoniazid resistance could lead to the emergence of MDR and XDR TB in places where these forms of TB are not currently a major problem. The need for IPT is currently greatest in countries that have the least capacity to deal with MDR and XDR TB. Although data on the impact of IPT on Isoniazid resistance is scanty and suggests that in the short term expansion of Isoniazid resistance may not be a major outcome of IPT [20], some modeling studies suggest that the widespread use of IPT as a public health intervention will reduce the incidence of tuberculosis in the short term but also will speed up the development of drug resistant TB [21]. It is therefore critical that the roll out of IPT programmes is carefully monitored for effectiveness and impact on isoniazid resistance.

## Intensified TB case finding

When screened for TB up to 25% of people living with HIV will have prevalent TB and an additional 11% will have incident TB. Persons initiating ART who develop TB have an increased risk of death which may reach 15–20%. The intensification of TB case finding through screening is likely to improve the safety of ART initiation and may improve the uptake of IPT [22]. Screening programmes have, however, varied and include symptom and sign screening, chest x-ray, sputum examination (for both smear microscopy and TB culture) and tests for latent TB such as the tuberculin skin test and or the interferon gamma release assays. In most situations screening tools appear to target pulmonary TB and may be unsuitable for use in children and for extrapulmonary forms of TB. There is a need for some standardization of TB screening tools which incorporate the most sensitive and specific constellation of symptoms, signs and tests for all forms of TB in all age groups. Regardless of algorithm, TB screening is vital. Tuberculosis is curable and linking persons living with HIV to TB diagnosis and DOTS-based treatment is potentially life saving for the individual and also an important public health measure.

## TB infection control

The places where people living with HIV receive care and treatment, including non medical care such as counseling centres and support group meeting places, may pose substantial risk for the transmission of TB. In poor countries of Sub-Saharan Africa hospitals wards and clinics may be very congested and can be fertile grounds for TB transmission. It is essential that simple but effective measures for preventing TB transmission in health care and other congregate settings are put in place especially now that MDR and XDR TB are increasingly becoming major concerns. Although TB Infection control guidelines for resource limited settings have been in place since 1999[23,24], WHO has recently developed 10 infection control actions with the slogan 'Safety without Stigma' that are intended to simplify TB infection control and also to reduce unwarranted stigma that may inadvertently be a consequence of TB infection procedures. Thus community engagement has been identified to be an essential first step in the development and implementation of an infection control plan (Table 2). It is anticipated that the approach advocated by WHO will reduce the hierarchical confusion that is at times observed in countries where health care workers demand state of the art technologies for infection control while ignoring basic administrative procedures such as prompt identification and diagnosis of potentially infectious patients. It will be essential to develop a set of key outcome indicators that infection control implementers can monitor to determine the effectiveness of the measures put in place.

**Table 2:** Ten essential actions for TB infection control

Involve community
Develop infection control plan

Safe sputum collection

Cough hygiene

Triage TB suspects

Rapid diagnosis and treatment

Improve ventilation

Protect Health care workers

Capacity building

Monitor infection control practices

## Challenges to TB/HIV care

### Initiating ART in HIV infected TB patients

The clinical care of HIV infected TB patients and in particular the treatment of these persons with life saving anti-retroviral medications is constrained by a number of factors. The provision of ART to HIV infected TB patients is intended to prevent further morbidity, mortality and to improve quality life. If ART is delayed patients may die before treatment is provided. Yet at the same time if treatment is given early during TB treatment, issues of pill burden, co-toxicities, the immune reconstitution inflammatory syndrome (IRIS) and pharmacokinetic interactions may contribute to morbidity or even mortality and render this therapy ineffective.

In Malawi it has been documented that the initiation of ART after the two months intensive phase of anti-TB treatment, as recommended by the ART programme in that country, did not decrease the unacceptably high mortality of HIV infected TB patients during the TB treatment [25]. However early initiation of ART within the first one month of anti-TB treatment may be a risk factor for TB-IRIS [26]. Although the majority of patients who experience TB-IRIS have mild disease, some episodes can be severe and end fatally. TB-IRIS therefore has implications for the way care is organized and demands robust referral systems. Ongoing studies examining the issue of optimal timing of ART initiation are expected to shed light on this important issue. Current clinical experience however suggests that ART should not be delayed on account of avoiding IRIS.

### Anti-TB drug induced hepatitis (DIH)

It is known that populations at risk for HIV and TB are also at risk for hepatitis B and or C infection. These hepatic infections may influence the risk of drug induced hepatis (DIC). HIV has been found to be an independent risk factor for DIH [27,28] which may be augmented by hepatitis C but not hepatitis B [29]. These hepatic reactions, although relatively uncommon, may complicate the provision of appropriate care for HIV infected TB patients leading to greater health resource utilization and a greater demand for secondary or tertiary level care. The weak health care systems in the settings where the TB/HIV disease burden is greatest may find this a tough issue to cope with.

## New threats to TB/HIV care

### Multi and extensive drug resistant TB and HIV

Unfortunately the progress being made in providing care and treatment to HIV infected TB patients is being threatened by the emergence of drug resistant TB and in particular multi and extensive drug resistant TB. On World Stop Day, 2006, the United States Centres for Disease Control and Prevention (CDC) published in the Morbidity and Mortality Weekly Report, a report that indicated the presence of extensive drug resistant TB (TB bacilli resistant to a fluroquinolone and one of the second line injectable drugs in addition to resistance to rifampicin and isoniazid) in virtually all regions of the world [30]. Of the 17,690 TB isolates examined between 2000–2004 from a network of international reference laboratories, 20% were MDR and 2% were XDR. Additionally it was reported that of the TB isolates from the United States (for 1993–2004), Latvia (for 2000–2002), and South Korea (for 2004), 4%, 19%, and 15% of MDR TB cases, respectively, were XDR.

This report was followed soon after by a disturbing report from Kwa Zulu Natal in South Africa. In the Kwa Zulu report, it was reported that of 542 patients with culture positive TB, 221(41%) had MDRTB. Of the 221 patients with MDRTB 53(24%) had XDRTB. Of the 53 patients with XDRTB, 52 (98%) died in a median 16 days after sputum collection. All 44 patients with XDR TB tested for HIV were HIV positive. From genotyping data the XDR TB isolates were found to be related and suggested common sources of infection. Over half of the patients had not been treated for TB previously and nearly two thirds of them had been admitted to hospital in the last two years [31]. This suggested that there was significant transmission of MDR and XDR TB and that the transmission was probably taking place in the hospital setting. The issue of infection control was sharply brought into focus as was the vulnerability of the HIV infected person to acquiring drug resistant TB at health care and other congregate settings and to progress rapidly to fatal disease. Institutional outbreaks of drug resistant TB affecting HIV infected persons are, however, not a new phenomenon. In the eighties and nineties such outbreaks were reported from several hospitals in the industrialized world. These outbreaks were associated with high mortality exceeding 70% [32].

The observed link between HIV and drug resistant TB is also not limited to Sub-Saharan Africa. Countries of the former Soviet Union have the highest rates of drug resistant TB and some of that resistance is associated with HIV [33]

In February this year, WHO released the fourth global drug resistance report which highlights the growing problem of anti-TB drug resistance. Among new cases any resistance was estimated at 17.0% (95 CLs 13.6–20.4), Isoniazid resistance at 10.3% (95% CLs 8.4–12.1) and MDR TB at 2.9 (95% CLs 2.2–3.6). In previously treated cases any resistance was estimated at 35.0% (95 CLs 24.1–45.8), Isoniazid resistance at 27.7% (95% CLs 18.7–36.7) and MDR TB at 15.3% (95% CLs 9.6–21.1). The global estimate is that is that there were 489,139 (95% CLs 455,093–614, 215) incident cases of MDR TB in 2006. As for XDR-TB, 45 countries have reported at least one case [34].

Drug resistant TB is a man made problem and is primarily a consequence of sub optimal TB control. Drug resistant TB has brought to the fore key challenges to TB care and prevention. These difficult to treat forms of TB are calling for the finding, as a matter of urgency, of new medicines that work and new and rapid diagnostics for both susceptible and drug resistant TB. Drug resistant TB has also brought to the fore ethical challenges such as the justification of isolation to protect communities without violating individual rights and the appropriateness of Direct Observation of Drug Intake (DOT) to ensure all anti-drug doses are ingested [35].

Prevention remains the best strategy and therefore the strengthening of TB control programmes to achieve high TB case finding and treatment success cannot be overemphasized. Closely linked to this is the institution of simple, affordable and effective measures to prevent TB transmission in health care and other congregate settings.

We conclude by emphasizing that TB/HIV is a major public health concern which demands a greater effort from TB and HIV prevention and care programmes, multinational technical agencies, financiers, researchers, communities and others. The added effort should see more rapid progress made in decreasing the burden of HIV in TB patients (testing, CPT and ART) and the burden of TB in PLHIV. Tuberculosis is appropriately gaining increasing importance to the HIV community. The 'Three I's' have also gained increasing importance among major opinion leaders. It is increasingly being recognized that if the HIV community does not own and provide leadership in the implementation of the 'Three I's', it is likely that TB will continue to be the number one killer of persons living with HIV and a persistent threat to the gains being made in improving access to HIV care and treatment.
